# Multimodal imaging shows acute multilayered retinal hemorrhages in heatstroke–a rare case report

**DOI:** 10.3389/fmed.2024.1322126

**Published:** 2024-02-20

**Authors:** Yi Zhang, Chunyan Lei, Xi Huang, Meixia Zhang

**Affiliations:** ^1^Department of Ophthalmology, West China Hospital, Sichuan University, Chengdu, China; ^2^Research Laboratory of Macular Disease, West China Hospital, Sichuan University, Chengdu, China

**Keywords:** heatstroke, multilayered retinal hemorrhage, preretinal hemorrhage, intraretinal hemorrhage, multimodal imaging

## Abstract

**Background:**

Heatstroke is a life-threatening disease clinically characterized by central nervous system dysfunction, multiorgan failure, and extreme hyperthermia. There are no reports about eye involvement in heat stroke. Here, we report a rare case of multilayered retinal hemorrhages in a patient with heatstroke.

**Case presentation:**

A 55-year-old male with a one-month history of blurry vision in both eyes presented at our department after suffering from heatstroke. His visual acuity was 5/20 OD and 10/20 OS. Fundus examination revealed retinal hemorrhages in both eyes. Fundus autofluorescence images and near-infrared reflectance images revealed well-defined retinal lesions. Optical coherence tomography helped to accurately locate the different layers of the lesions, including the nerve fiber layer, sub-inner limiting membrane, outer plexiform layer, ellipsoid zone and Henle fiber layer hemorrhages. We followed up with the patient for 8 months. At the last follow-up, his visual acuity was 20/20 in both eyes, and fundus examination showed that retinal hemorrhages were almost completely absorbed.

**Conclusion:**

To our knowledge, this is the first report on multilayered retinal hemorrhages secondary to heat stroke. Intraretinal and preretinal hemorrhages can gradually resolve, and the patient’s vision will improve with the absorption of the retinal hemorrhages. Multimodal imaging may help to reveal additional details about retinal lesions and monitor the course of the disease.

## Introduction

Heatstroke is a life-threatening disease clinically characterized by central nervous system dysfunction, multiorgan failure, and extreme hyperthermia (1, 2). Complications of heatstroke include complications involving the central nervous, cardiovascular, pulmonary, and gastrointestinal systems, and there is often renal and hematologic involvement (2). However, there are no reports about eye involvement in heat stroke. Here, we report a rare case of heatstroke with multilayered intraocular hemorrhages.

## Case report

A 55-year-old male with a one-month history of blurry vision in both eyes presented at our department after suffering from heatstroke. One month prior, the patient did outdoor work on a hot (ambient temperature up to 40°C) and humid day. Approximately 3 h later, he developed a consciousness disorder. He was immediately transported to the neurological intensive care unit and diagnosed with heat stroke. The vital signs of this patient were checked. His body temperature was 39.8°C, his blood pressure was 111/59 mmHg, his heart rate was 159 beats/min, and his oxygen saturation was 92%. Subsequently, the comatose patient suffered from generalized epileptic seizures, gastrointestinal hemorrhage and respiratory insufficiency. His blood pressure reduced to 72/39 mmHg. Laboratory tests revealed anemia (hemoglobin 106 g/L), decreased platelet count (15 × 10^9^/L), elevated lactic dehydrogenase (LDH 410 IU/L), elevated D-dimer (1.26 mg/L), and positivity for fecal and urinary occult blood (the normal ranges are usually 130–175 g/L for hemoglobin, 100–300*10^9^/L for platelets, 120–250 IU/L for LDH and < 0.5 mg/L for D-dimer). Computed tomography (CT) of the brain was performed, and no specific parenchymal lesions were detected. Immediate cooling was initiated by applying cooling blankets to the patient and putting ice packs to the patient’s head, neck, and groin. The patient was also infused with cold normal saline, and iced gastric and colonic lavage were performed. In addition, he received treatment, including oxygen inhalation, replenishment of platelets, and support of his airways, breathing, and circulation. Treatments to control seizures, improve blood pressure and organ perfusion, and manage complications were also given to this patient. Antibiotic treatment was given. The patient regained consciousness 8 days after the onset of coma. Since this patient regained consciousness, he complained of blurry vision in both eyes, but he did not receive any special ophthalmological treatment. He had no history of diabetes, hypertension, systemic vascular disease, inflammatory disease, ocular discharge or ocular trauma. His family history was also unremarkable.

**Figure 1:**
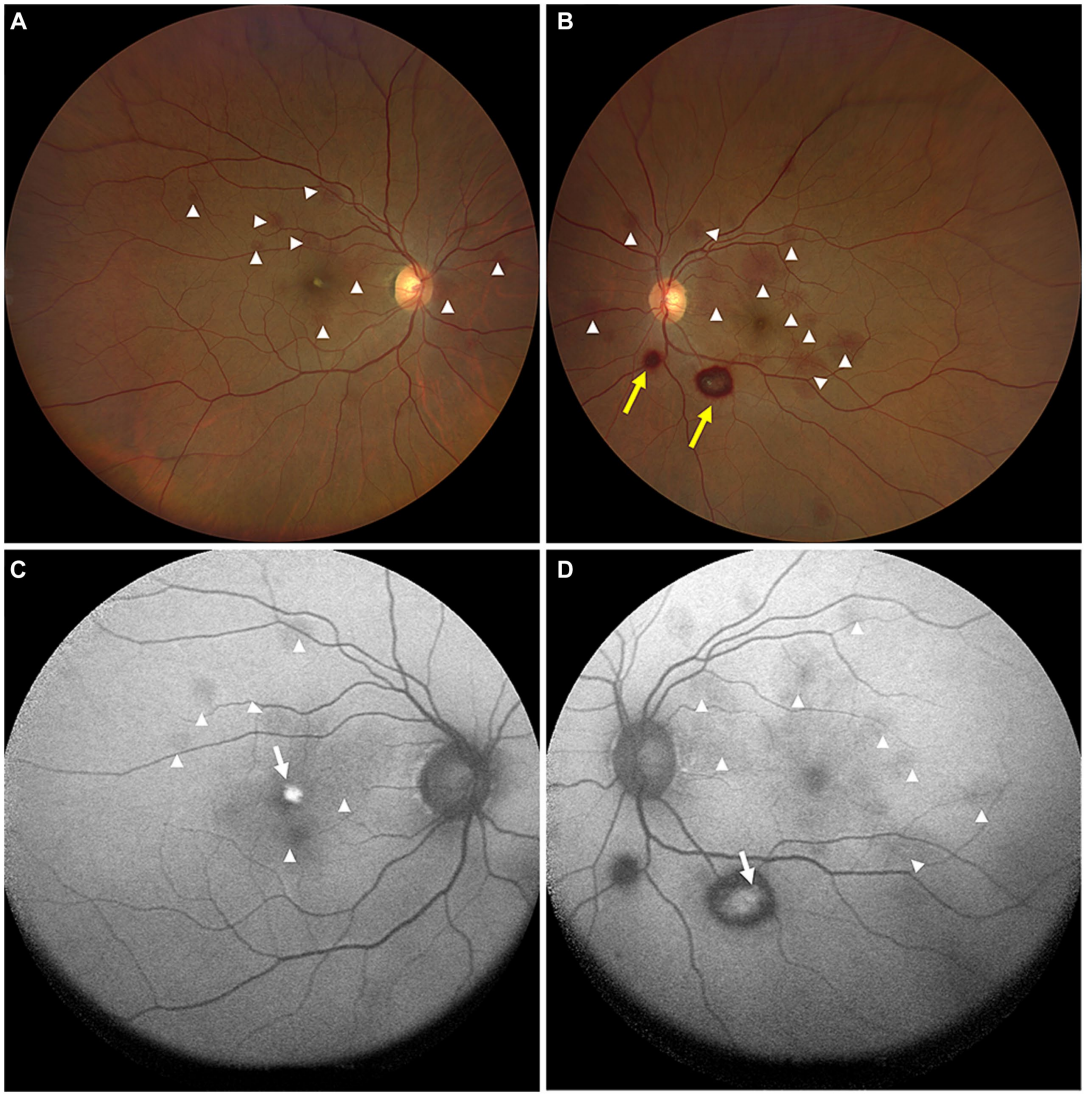
Color ophthalmoscopic images of both eyes **(A,B)** showing several reddish and yellowish lesions in different layers of retina. The lesions included preretinal hemorrhages (yellow arrows) and intraretinal hemorrhages (white arrowheads). FAF images **(C,D)** of both eyes revealed the hemorrhagic retinopathies as hyper-FAF (white arrows, representing partial absorption of retinal hemorrhages) and hypo-FAF (white arrowheads, representing fresh retinal hemorrhages).

**Figure 2:**
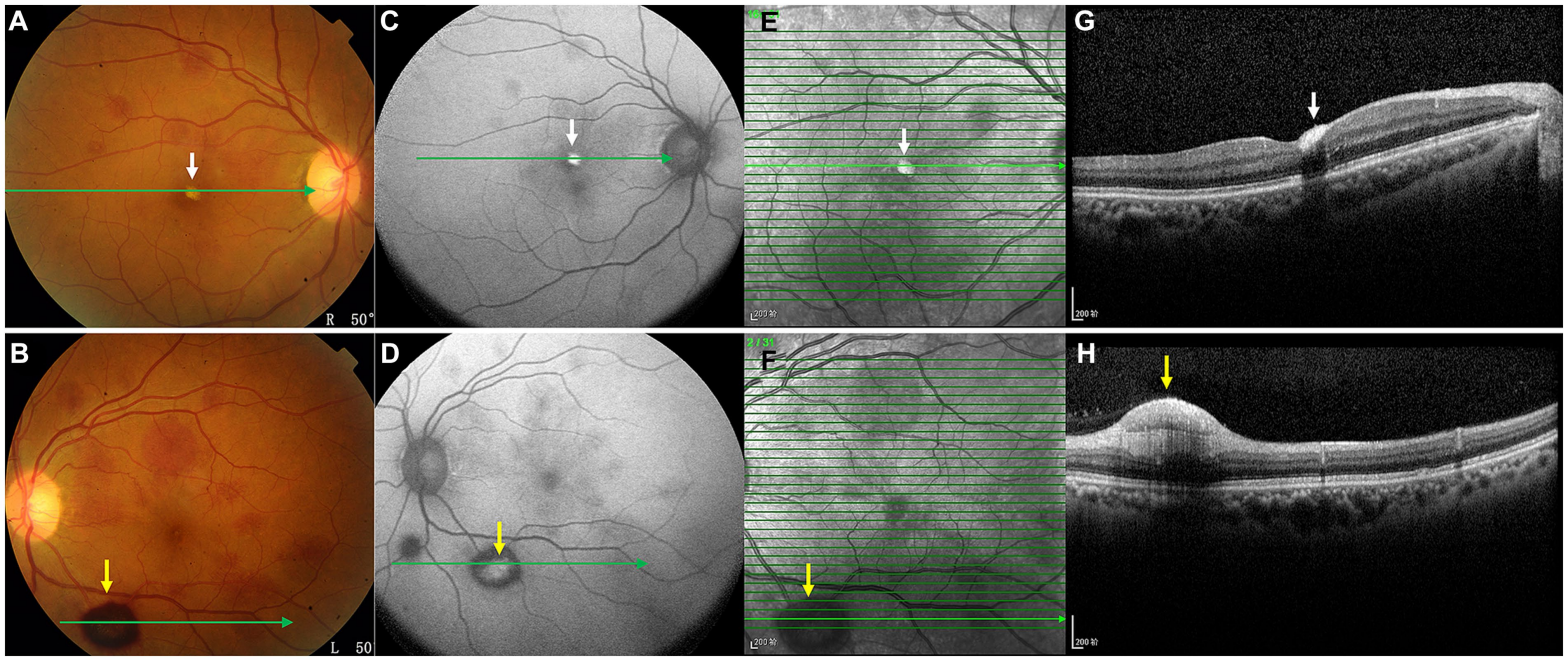
OCT images (**G,H**, arrows) of the hemorrhagic lesions shown in the color ophthalmoscopic **(A,B)**, FAF **(C,D)**, and NIR **(E,F)** images. OCT through the lesions revealed dense hyperreflective hemorrhages of the RNFL (**G**, white arrow) and sub-ILM (**H**, yellow arrow).

**Figure 3:**
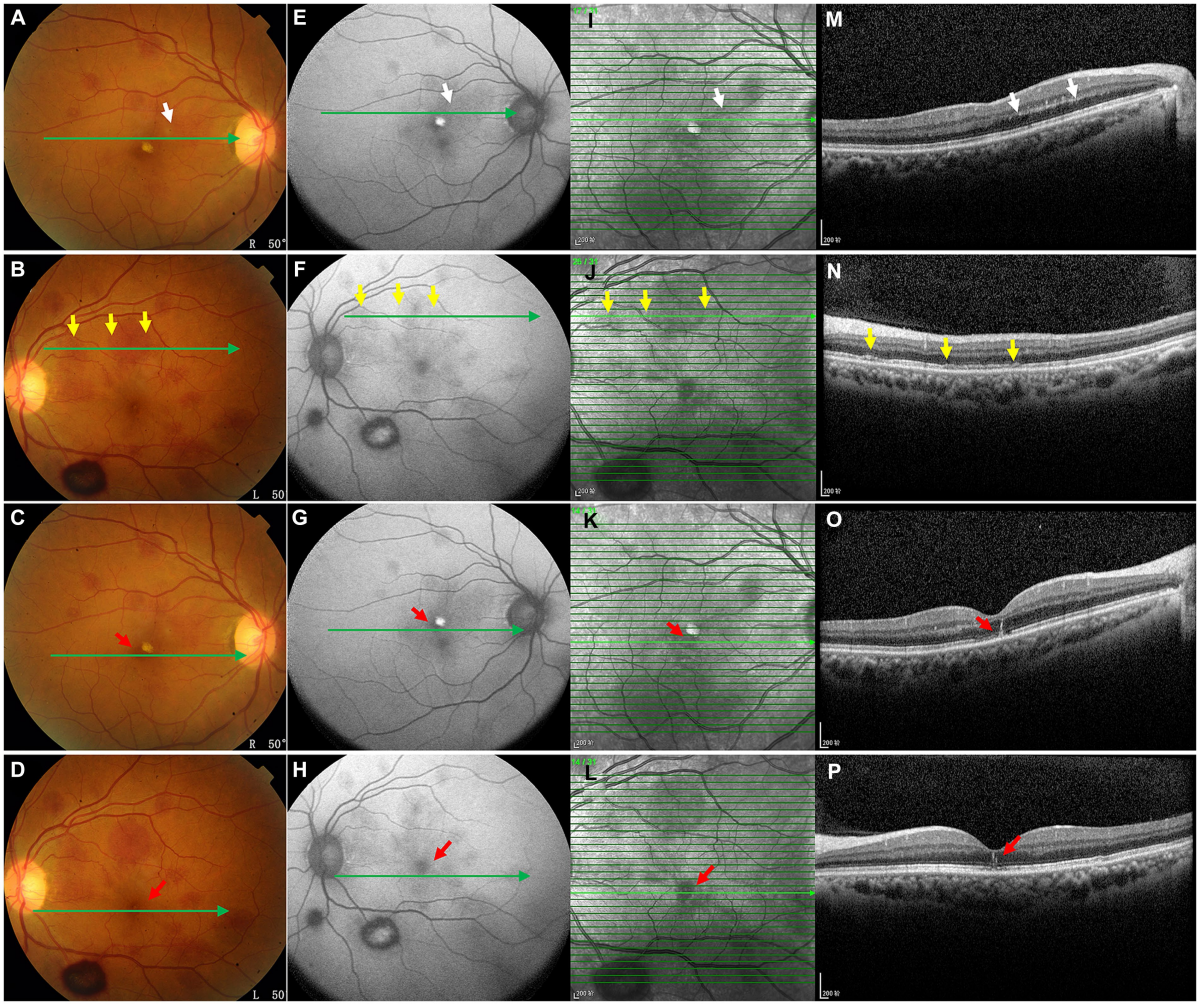
OCT images **(M–P)** of the lesions shown in the color ophthalmoscopic **(A–D)**, FAF **(E–H)** and NIR **(I–L)** images. OCT through the lesions revealed a hyperreflective band at the posterior border of the OPL (**M**, white arrows) and attenuation of the EZ (**N**, yellow arrows), representing hemorrhages of these outer retinal layers. OCT also showed EZ disruption and HFL hemorrhages (**O,P**, red arrows).

**Figure 4:**
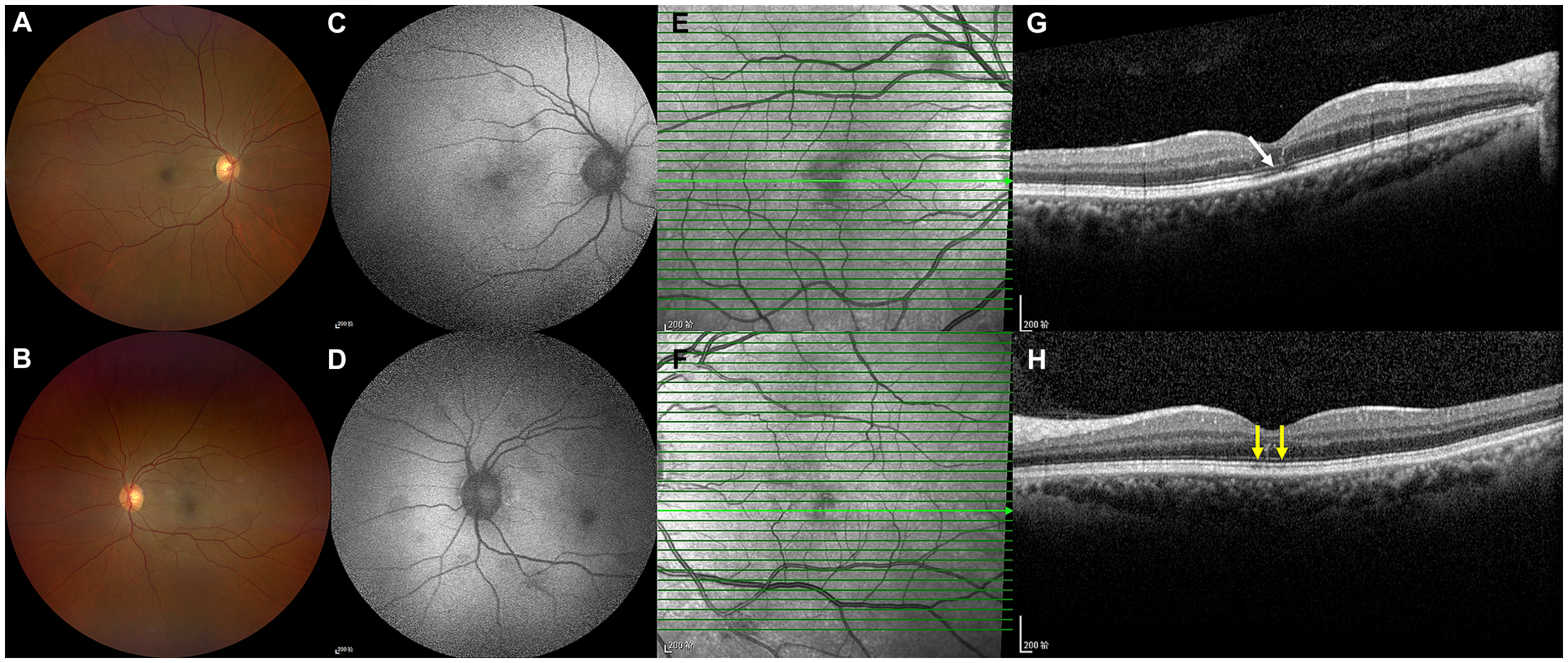
Color ophthalmoscopic images of both eyes were normal **(A,B)**. However, FAF images **(C,D)** and NIR images **(E,F)** revealed that mild retinal lesions remained. OCT **(G,H)** revealed slight changes in the outer retinal layers. The continuities of the EZ (**G**, white arrow) and interdigitation zone (**H**, yellow arrows) were not fully recovered.

On the current examination, his visual acuity was 5/20 OD and 10/20 OS. The patient’s intraocular pressure was 11.5 mmHg OD and 11.1 mmHg OS. The anterior segment examination was unremarkable. Fundus examination revealed multilayered retinal hemorrhages in both eyes (Figures 1A,B, 2A,B, 3A–D), including preretinal hemorrhages and intraretinal hemorrhages. On his fundus images, we could see that his retinal hemorrhages were mainly distributed around the macular region and optic disk. The preretinal hemorrhages were dark red, while the intraretinal hemorrhages were light red. All retinal hemorrhages appeared as circular patches, with a size between 1/5–1 papillary diameter. Fundus autofluorescence (FAF) images of both eyes revealed that the lesions were either hyper-FAF or hypo-FAF (Figures 1C,D, 2C,D, 3E–H), representing different periods of hemorrhages. Near-infrared reflectance (NIR) images showed well-defined retinal lesions (Figures 2E,F, 3I–L). Optical coherence tomography (OCT) helped to accurately locate the different layers of the lesions, including the retinal nerve fiber layer (RNFL) (Figure 2G), sub-inner limiting membrane (sub-ILM) (Figure 2H), outer plexiform layer (OPL) (Figure 3M), ellipsoid zone (EZ) (Figure 3N) and Henle fiber layer (HFL) (Figures 3O,P) hemorrhages. Laboratory tests revealed that his hemoglobin level was 131 g/L, LDH was 196 IU/L and platelet count was 128*10^9^/L. The laboratory test results were normal, and his retinal hemorrhages were partially absorbed. We did not administer any special treatment except for observation. At the follow-up visit 8 months later, his visual acuity was 20/20 in both eyes, and fundus examination showed that his retinal hemorrhages were almost completely absorbed (Figures 4A,B). However, FAF (Figures 4C,D) and NIR (Figures 4E,F) imaging helped to detect that mild retinal lesions were remained. OCT through the macula also revealed changes in the outer layers of the retina (Figures 4G,H). The continuities of the EZ (Figure 4G) and interdigitation zone (Figure 4H) were not fully recovered.

## Discussion

Heatstroke is a hazardous condition and may cause multiple-organ dysfunction (1, 3). This is the first case report of hemorrhagic retinopathy as a complication of heat stroke. In this case, multilayered retinal hemorrhages were notable. Retinal hemorrhages are an important sign of an underlying systemic vascular disorder and can provide insight into its etiology. We detected hemorrhages at various levels of the retina, including preretinal, sub-ILM, RNFL, OPL, EZ, and HFL. There are four different capillary networks in the retina: the RNFL, retinal ganglion cell layer, border of the inner plexiform layer (IPL) and superficial boundary of the inner nuclear layer (INL), and boundary of the deep INL and OPL (4). The multilayered retinal hemorrhages in this case were caused by multilayered vasculopathies. The mechanism of multilayered vasculopathies is likely multifactorial. First, heatstroke is the most hazardous condition in a spectrum of illnesses progressing from heat exhaustion to heat injury and heat stroke, in which a shared finding is hyperthermia (3). A continued increase in the core body temperature can lead to an increase in cardiac output. When the cardiac output is insufficient to cope with high thermoregulatory needs, it leads to a direct cytotoxic effect and an inflammatory response (5). Increased cardiac output, cytotoxic effects and inflammatory responses may cause vascular endothelial damage and subsequent hemorrhage (5). In our case, the patient developed multiorgan damage and hemorrhages due to core temperature disturbances, which can cause heat-induced widespread microthrombosis, inflammatory injury and hemorrhage (3). Second, this patient also experienced severe platelet reduction. Heatstroke can cause hematologic disturbances, including low platelet counts (6). Coagulopathy is a common complication of severe heatstroke, and thrombocytopenia may be associated with disseminated intravascular coagulation (DIC) (3). Heatstroke patients may experience hemorrhagic complications because of excessive consumption of platelets. In addition, anemia is an important risk factor for multilayered hemorrhages (7, 8). Studies have shown that fundus lesions could be accompany symptoms in many hematological diseases and that anemia combined with thrombocytopenia increases the frequency of retinal hemorrhages (8, 9). Third, we speculated that hypoxia of retina related to anemia, hypotension and respiratory insufficiency may cause vasodilatation and abnormal vessel leakage, thus leading to retinal hemorrhages. In our case, the patient’s retinal hemorrhages, which were caused by heatstroke, should be distinguished from retinal hemorrhages caused by other diseases, such as diabetes, hypertension, hematological diseases, trauma, intracranial diseases, systemic lupus erythematosus, Behcet disease, etc. Retinal hemorrhages can also be caused by medications, such as anticoagulants. This patient was healthy before and had not taken any medications. His laboratory test results were normal when he recovered. Based on this, we believe that the patient’s retinal hemorrhages were caused by heatstroke.

In addition to hemorrhagic retinopathies, foveal EZ disruption was also detected by OCT. We speculated that the attenuated EZ in our patient might be related to ischemia of the deep capillary plexus (DCP), which is located in the outermost portion of the INL. The severe hypotension, anemia and respiratory insufficiency in this patient may have led to ischemia of the DCP and subsequently to lesions of the foveal EZ. Recent studies have reported that the DCP is the major level of venous outflow and may be at greatest risk of injury and disruption secondary to acute changes in venous pressure (10). Venous pressure changes secondary to heat stock may be the main reason for the outer layer retinal lesions in our case. In addition, retinal hemorrhages can also cause retinal photoreceptor damage. Based on the observation of this patient, we found that the changes of EZ lasted longer than the retinal hemorrhages.

With the advent of more sophisticated imaging systems, we can detect more detailed retinal lesions. In this case, OCT helped us locate the different layers of hemorrhagic lesions, which cannot be identified by color ophthalmoscopic images. FAF and NIR images revealed the morphology and additional details of the lesions. With the absorption of retinal hemorrhages, multimodal imaging can help to identify mild lesions and follow up on them. In this patient, the macular hemorrhages may be the main reason of his decreased vision. We followed up with the patient for 8 months and observed that the intraretinal and preretinal hemorrhages were mostly absorbed, and his vision had improved.

## Conclusion

To our knowledge, this is the first report on multilayered retinal hemorrhages secondary to heatstroke. This case study emphasized the necessity of fundus examination in heat shock patients and reminded us that hemorrhagic retinopathy and ischemic retinopathy may coexist in heatstroke patients. Most intraretinal and preretinal hemorrhages can be absorbed, and vision can improve with the absorption of retinal hemorrhages. Multimodal imaging can be used to reveal additional details about retinal lesions.

## Data availability statement

The original contributions presented in the study are included in the article/supplementary material, further inquiries can be directed to the corresponding author.

## Ethics statement

Written informed consent was obtained from the individual(s) for the publication of any potentially identifiable images or data included in this article.

## Author contributions

YZ: Data curation, Formal analysis, Investigation, Project administration, Writing – original draft, Writing – review & editing. CL: Investigation, Supervision, Writing – review & editing. XH: Investigation, Supervision, Writing – review & editing. MZ: Data curation, Resources, Supervision, Writing – review & editing.
